# Disentangling effects of multiple stressors on matter flow in a lake food web

**DOI:** 10.1002/ece3.7789

**Published:** 2021-06-21

**Authors:** Shuran Cindy Wang, Xueqin Liu, Yong Liu, Hongzhu Wang

**Affiliations:** ^1^ State Key Laboratory of Freshwater Ecology and Biotechnology Institute of Hydrobiology Chinese Academy of Sciences Wuhan China; ^2^ Key Laboratory of Water and Substrate Sciences (Ministry of Education) College of Environmental Sciences and Engineering Peking University Beijing China

**Keywords:** biological invasion, biomanipulation, ecosystem functioning, ecosystem management, eutrophication, scenario forecasting

## Abstract

Understanding the relative importance of multiple stressors is valuable to prioritize conservation and restoration measures. Yet, the effects of multiple stressors on ecosystem functioning remain largely unknown in many fresh waters. Here, we provided a methodology combining ecosystem modeling with linear regression to disentangle the effects of multiple stressors on matter flow, an important ecosystem function. Treating a shallow lake as the model ecosystem, we simulated matter flow dynamics during 1950s–2010s with different combinations of stressors by Ecopath with Ecosim (EwE) modeling and determined the relative importance of each stressor by generalized linear mixed models. We found that matter flow of the lake food web was highly dynamic, attributing to effects of multiple anthropogenic stressors. Biological invasion played the strongest role in driving the matter flow dynamics, followed by eutrophication, while biomanipulation (i.e., phytoplankton control by planktivorous fish stocking) was of little importance. Eutrophication had a stronger role on primary producers, pelagic food chain, and top predators, while biological invasion on consumers in the middle of food chains. The former was more important in driving the quantity of matter flow, while the latter on trophic transfer efficiencies. Scenario forecasting showed that reducing nutrients contents could largely shape the matter flow pattern, while biomanipulation had little effect. Our findings provided new insights into understanding the mechanistic links between anthropogenic stressors and ecosystem functioning by combining ecosystem modeling with linear regression.

## Graphical abstract

A methodology combining ecosystem modelling with linear regression was provided. The relative importance of multiple stressors on matter flow in the lake food web was determined. Eutrophication had a stronger role on the quantity of matter flow, while biological invasion was more important on trophic transfer efficiency.

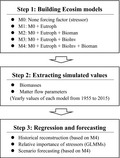

## 1 INTRODUCTION

Freshwater ecosystem is probably the most endangered ecosystem of the world. The past decades have seen a continuous decline in biodiversity and ecological integrity in fresh waters, at a pace much faster than that for land and sea (Bunn, 2016; WWF, 2018). A number of anthropogenic stressors, including water pollution, flow modification, habitat degradation, overexploitation, and biological invasion, have been recognized as major stressors across the globe (Dudgeon et al., 2006). These stressors are likely to be intensified in most freshwater ecosystems in the next decades, and new threats continue to emerge in recent years, posting persistent challenges for conservation science, initiatives, and management (Settele et al., 2014, Reid et al., 2019). Although the effects and underlying mechanisms of anthropogenic stressors have been well documented for biological communities, our understanding of their impacts on ecosystem functioning (e.g., matter and energy flows) remains very limited (Soranno et al., 2019; Woodward & Perkins, 2015). To fill this knowledge gap is undoubtedly important and helpful for conservation and restoration of freshwater ecosystems, considering the needs of a holistic conservation strategy and ecosystem‐based management (Bunn, 2016; Goulding et al., 2019).

One major challenge is to disentangle the effects of multiple stressors on ecosystem functioning in fresh waters. It has long been recognized that environmental stressors can interact to exacerbate biodiversity loss and ecological degradation (Jackson et al., 2016; Ormerod et al., 2010). These stressors can occur simultaneously or sequentially, generating complex effects that might be antagonistic, synergistic, additive, or reversed (Jackson et al., 2016). Consequently, it is essential to explore the net effects of one stressor while removing the effects of others considering conservation and restoration of degraded freshwater ecosystems. Yet, empirical assessment of net effects of multiple stressors on ecosystem functioning is rarely documented in fresh waters, although there is an increasing concern on the “multiple stressor” problem in the last decades (Jackson et al., 2016; Reid et al., 2019). Disentangling the effects of multiple stressors in real freshwater ecosystems faces logistical and ethical obstacles related to spatial‐temporal scale, data quality, and replication of tests (Galic et al., 2018). To cope with these obstacles, a feasible strategy is to reconstruct the ecosystem dynamics under environmental stressors by predictive models (Galic et al., 2018).

Previously, freshwater ecosystem functioning has been usually addressed at single trophic levels, with much attention being paid on biological production (e.g., primary and secondary) and decomposition (Handa et al., 2014; Low‐Décarie et al., 2014). Yet, ecosystem functioning across multiple trophic levels, such as matter and energy flows in food webs, has been poorly explored in many fresh waters (Barnes et al., 2018; Woodward & Perkins, 2015). Not only are matter and energy flows themselves important ecosystem functions, but also they reflect aggregate processes across multiple trophic levels (Barnes et al., 2018). Therefore, the flow of matter or energy through food web can be used as an index of ecosystem multifunctionality, providing a common currency for cross‐ecosystem comparisons (Barnes et al., 2018). Although the concept and importance of matter and energy flows have long been acknowledged, quantification of these functions in real ecosystems remains challenging given the complexity and dynamicity nature of trophic networks (Ulanowicz et al., 2014). Determination of matter or energy flow through a given feeding link is based on ecological energetics, while estimation of the total flow through a food web usually needs a mass balance model methodology, such as Ecopath with Ecosim (Christensen & Walters, 2004; Tomlinson et al., 2014).

Lakes are ideal model systems for food web study as they are relatively closed and have clearly boundaries with adjacent ecosystems (McMeans et al., 2016). Here, we explored the effects of multiple stressors on lake ecosystem functioning applying an ecosystem modeling technique and statistical analyses. Treating a shallow highland lake as the model system, we have three specific objectives. First, we aimed to reconstruct historical patterns of matter flow in the lake food web from the 1950s to the 2010s. Second, we aimed to disentangle the effects of multiple stressors on matter flow patterns of the lake with emphasis on three key stressors, that is, eutrophication, biological invasion, and biomanipulation. Third, we aimed to explore the changes of matter flow under different management scenarios.

## 2 METHODS

### 2.1 The study lake

**FIGURE 1:**
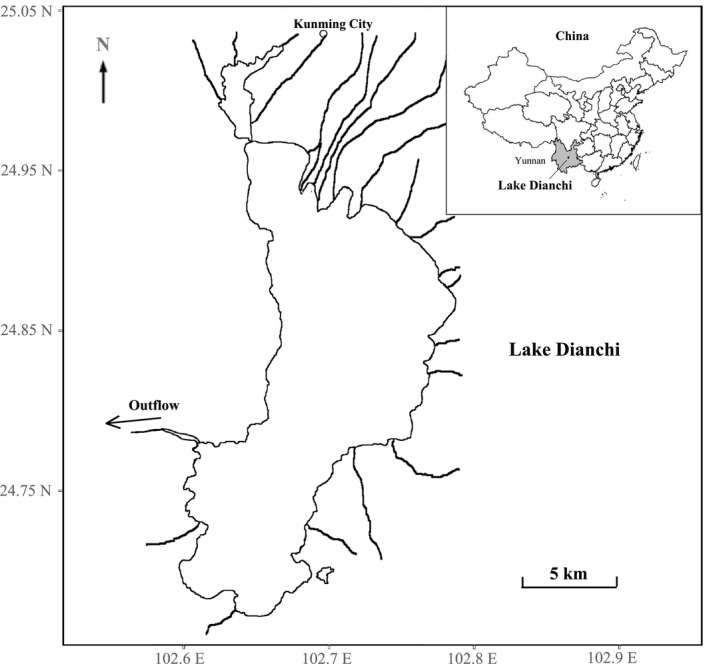
Location of the study lake, Lake Dianchi, China

In the present study, we selected a shallow plateau lake, Lake Dianchi, as the model ecosystem. The lake (24°40’~25°02’N, 102°36’~102°47’E) is located on the Yunnan‐Guizhou Plateau in the subtropical area of China, with an elevation of 1886.4 m (a.s.l.) (Figure 1). It is a large shallow lake, with a surface area of approximately 300 km^2^ and an average water depth of 4.4 m (Wang et al., 2018). The lake has suffered from a number of anthropogenic stressors since the late 1950s, resulting in serious degradation of the ecosystem (Li et al., 2014). The first stressor is eutrophication. The lake water quality has deteriorated rapidly since the 1980s attributing to continuous increase of nutrient loading, and its trophic state has shifted to eutrophic in the early 1990s (Li et al., 2014). At present, it is hypereutrophic, with the total nitrogen being 2.06 mg/L and total phosphorus 0.10 mg/L in offshore lake water during 2015–2016 (Wang et al., 2018). Heavy cyanobacteria blooms occurred in the lake every year after it was eutrophied. The second stressor is biological invasion. From 1958, a total of twenty‐nine species mainly from the lowland region of the Yangtze River have been introduced into the lake either intentionally or accidentally (He & Liu, 1985). The introduction of these non‐native species has exerted great detrimental effects on local fish communities, given that around 85% number of native species have disappeared from the lake (Chen et al., 2001). The third stressor is habitat loss. A total of 29.4 km^2^ of littoral area (ca. 10% lake surface area) of the lake have been reclaimed (i.e., transformed to land) which happened mainly in 1970 (Li et al., 2014). The fourth stressor is biomanipulation. In purpose of phytoplankton control, stocking of pelagic filtering fishes, mainly silver carp (*Hypophthalmichthys molitrix* (Valenciennes)) and bighead (*Hypophthalmichthys nobilis* (Richardson)), has been carried out every year since the early 2000s. The amount of stocking fish varied from ca. 20–100 tonnes among years.

### 2.2 Model construction

We applied the Ecopath with Ecosim (EwE, version 6.2, www.ecopath.org) modeling framework to estimate matter flow, more specifically biomass flow, through the lake food web. The EwE is a trophic mass balance modeling suite, containing three components, that is, Ecopath for a static module, Ecosim for a time dynamic module, and Ecospace for a spatial and temporal dynamic module. The basic equation for the Ecopath model is expressed as:

(1) $$B_{i}{\left(\frac{P}{B}\right)}_{i}EE_{i}‐\sum_{j=1}^{n}B_{j}{\left(\frac{P}{B}\right)}_{j}DC_{\mathrm{ij}}‐Y_{j}‐E_{j}‐BA_{j}=0$$

where *DC_ij_* is the fraction of resource *i* in the diet of consumer *j*, *B* is biomass; *P/B* is production to biomass ratio, *EE* is ecotrophic efficiency, *Y* is total fishery catch rate, *E* is net migration rate, and *BA* is biomass accumulation rate.

Ecosim provides predictions of biomass and harvest rates of each functional group with parameters inherited from the Ecopath model. The equation for Ecosim model is as follows:

(2) $$\frac{dB_{i}}{\mathrm{dt}}=g_{i}\sum_{j}Q_{\mathrm{ji}}‐\sum_{j}Q_{\mathrm{ij}}+I_{i}‐\left(MO_{i}+F_{i}+e_{i}\right)B_{i}$$

where $\frac{dB_{i}}{\mathrm{dt}}$ is the rate of change in biomass of functional group *i*, *g_i_* is the net growth efficiency (i.e., production to consumption ratio). *Q_ji_* is the consumption rates by group *i*, calculated based on “foraging arena” concept (Ahrens et al., 2012). *Q_ij_* is the predation mortality rate on group *i*, *MO_i_* is the nonpredation natural mortality rate, *F* is fishing mortality rate, *e* is emigration rate, and *I* is immigration rate.

In a previous study, we constructed a high‐resolution (mainly based on species‐level data) static Ecopath model (hereafter Model2016) of the lake based on data of 2010–2016 (Wang et al., 2020). In the present study, we followed the Ecosim routine to conduct a temporal dynamic analysis during the 1950s–2010s, a time period covering major anthropogenic disturbances of Lake Dianchi. We collated biological data of major aquatic communities, including taxon composition, density, biomass, and fish catch, of the lake during 1950–2016 from various data sources (Table S1). Since there was no long‐term yearly data recorded for most biological communities, we compiled biological data for every decade (Table 1). Data of 1958 and 1959 were included in the dataset of the 1960s, considering that species introduction started from 1958 in Lake Dianchi (He & Liu, 1985). The dataset of 1950s was used to construct the initial Ecopath model, and the remaining datasets were used to test model predictions. Consequently, the time period for model prediction was set to be 1955–2015.

**TABLE 1:** Estimated biomass (dry mass, 10^3^kg/km^2^) and parameters of functional groups used for Ecosim model construction in Lake Dianchi

Input parameters	Piscivorous fish	Predatory fish	Omnivorous fish	Grazing fish	Planktivorous fish	Macroinvertebrates	Shrimps	Zooplankton	Macrophytes	Phytoplankton	Benthic algae
Biomass											
1950s	0.09	0.08	1.00	0.11	0.001	40.00	0.22	3.11	3,020	1.72	2.00
1960s	0.005	0.25	0.06	1.49	0.51	‐	2.34	‐	1,361	‐	‐
1970s	0.02	0.18	1.17	0.19	0.36	‐	3.24	‐	1,000	‐	‐
1980s	0.01	0.05	0.54	0.91	0.94	‐	1.85	43.69	420	0.21	‐
1990s	0.05	0.38	1.32	0.01	0.77	1.41	1.43	46.65	136	0.05	‐
2000s	0.21	0.36	0.90	0.02	0.79	2.22	1.58	10.39	266	11.39	‐
2010s	1.14	0.48	2.88	0.01	5.52	0.25	2.14	1.54	359	6.85	4.39
Parameter											
P/B	1.33	1.41	1.50	1.18	1.85	8.17	3.23	108.00	1.47	117.66	107.50
Q/B	5.88	9.09	20.56	73.21	9.61	42.42	12.50	800.00	0.00	0.00	0.00
MO	0.52	0.60	0.58	0.52	0.60	*N*/A	*N*/A	*N*/A	*N*/A	*N*/A	*N*/A

Note

‐, datum was unavailable. P/B, production to biomass ratio; Q/B, consumption to biomass ratio; MO, nonpredation natural mortality rate.

Food web components were classified into 12 functional groups, including 1 detritus group, 3 primary producer groups (phytoplankton, benthic algae, and macrophytes), 3 invertebrate groups (zooplankton, macroinvertebrates, and shrimps), and 5 fish groups (planktivorous, grazing, omnivorous, predatory, and piscivorous fish). To perform the initial model requires to input the following parameters: biomass, P/B ratio, Q/B (consumption/biomass) ratio, and diet composition of consumers. Biomass of fish as well as shrimps was estimated as the yield divided by fishing mortality according to Wang et al. (2020). Biomasses of other biological groups were estimated from the collated datasets. Wet biomass was converted to dry biomass according to dry/wet ratios (Wang et al., 2020, and the references therein). The P/B ratios, Q/B ratios, and diet composition of consumers were referred to the Model2016 (Table 2). If data were unavailable from the Model2016, values were cited from literature; otherwise, values of the same or most closely related genus were used.

**TABLE 2:** Diet composition (proportion) of functional groups used for Ecosim model construction in Lake Dianchi

Code	Prey \ predator	1	2	3	4	5	6	7	8
1	Piscivorous fish	0	0	0	0	0	0	0	0
2	Predatory fish	0.04	0	0	0	0	0	0	0
3	Omnivorous fish	0.85	0	0	0	0	0	0	0
4	Grazing fish	0.11	0	0	0	0	0	0	0
5	Planktivorous fish	0.00	0	0	0	0	0	0	0
6	Macroinvertebrates	0	0.70	0.60	0	0	0	0	0
7	Shrimps	0	0.30	0.10	0	0	0	0	0
8	Zooplankton	0	0	0.08	0	0.50	0	0	0
9	Phytoplankton	0	0	0.08	0	0.50	0	0	1.00
10	Macrophytes	0	0	0.03	0.20	0	0	0	0
11	Benthic algae	0	0	0.10	0.60	0	0.50	0.50	0
12	Detritus	0	0	0.01	0.20	0	0.50	0.50	0

Environmental stressors were added into the model as forcing factors to test their role in driving the matter flow dynamics. Three major stressors, that is, eutrophication, biological invasion, and biomanipulation, were considered. Habitat loss was not included since it happened within a short time period (Li et al., 2014) and hardly be treated as a continuous variable for model analyses. Each stressor was quantified, and the annual data were used. The content of total phosphorus (TP) in lake water was used as a surrogate of eutrophication according to previous studies (Schindler, 2012), and data were collated from literature (Li et al., 2014; Wang et al., 2018). Regarding biological invasion, introduced species were classified into five invasion groups, that is, predatory, planktivorous, grazing, omnivorous, and shrimp, and each group was quantified based on their catch information (amount and relative abundance) (Table S1). Regarding biomanipulation, the releasing amount of planktivorous fish was used for quantification (Table S1). Stressors were set to affect the feeding relationships among functional groups, and only the direct effects were considered for simplifying the model. Eutrophication (indicated by TP here) was set to exert a positive effect on phytoplankton biomass, and only its effect on phytoplankton was considered since the TP–phytoplankton relationship was relatively well studied in shallow lakes. Regarding biological invasion and biomanipulation, only the direct predation effects were considered based on their dietary composition (Table 2).

**FIGURE 2:**
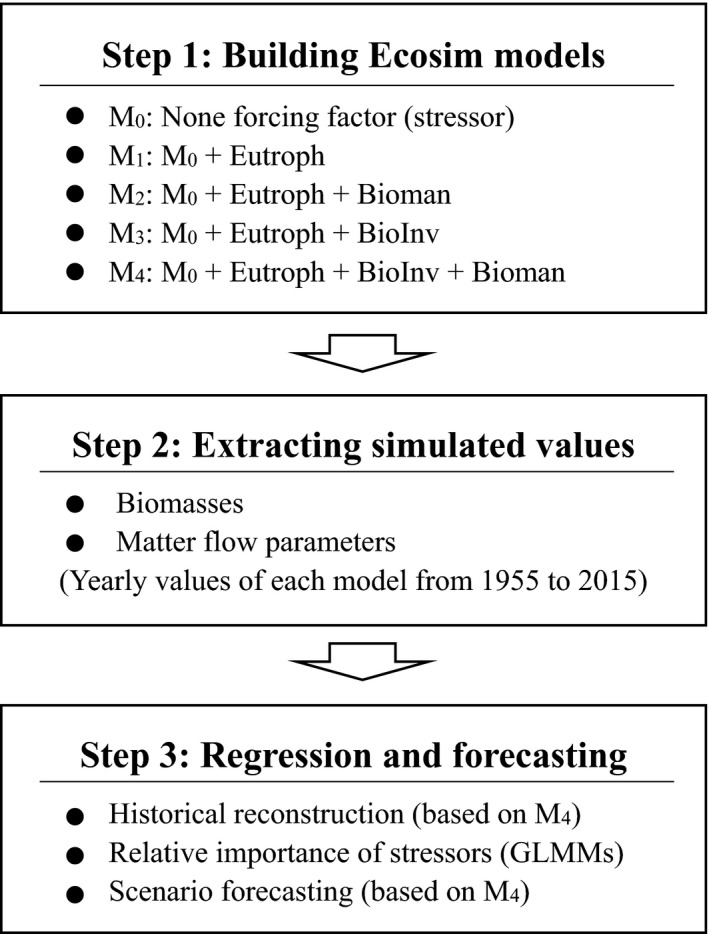
A conceptual diagram showing the methods combining Ecosim models with linear regression in the present study. Eutroph, eutrophication; BioInv, biological invasion; Bioman, biomanipulation; GLMMs, generalized linear mixed models

Five Ecosim models were constructed by adding different combinations of forcing factors (Figure 2: Step 1), that is, M_0_: none forcing factor, M_1_: + eutrophication, M_2_: + eutrophication + biomanipulation, M_3_: + eutrophication + biological invasion, and M_4_: + eutrophication + biological invasion + biomanipulation. Therefore, M_0_ represented a status without any anthropogenic disturbance, while M_4_ represented a status mostly close to reality and would be the best fitted among the five models. Model balancing and fitting were referred to common procedures in EwE modeling (Christensen & Walters, 2004). All Ecosim models were performed with a yearly timestep. Diet matrix was finely tuned to ensure that each model was balanced and stable over long period. The effect sizes of forcing factors on feeding relationships were also adjusted to ensure that the simulated biomasses matched with the observed data as much as possible. The fitting procedure was to reduce the sum of squares (SS) of differences between simulations and observations, and the fitted model was the one with the lowest SS value. According to Natugonza et al. (2020), the modeling efficiency index (MEF) was used to check how well the simulated values fitted to the observed data. Regarding the model M_4_, most simulations matched with the observations to an acceptable degree (Figure S1).

### 2.3 Data analyses

Simulated yearly data of biomasses and matter flow parameters were extracted from each Ecosim model output (Figure 2: Step 2). The following matter flow parameters were selected: total system throughput (TST), primary production required (PPR), Finn's cycling index (FCI), and average transfer efficiencies (TE) of total system (TE_total_), grazing food chains (TE_grazing_), and detritus food chains (TE_detritus_) (Christensen & Walters, 2004). TST describes the total matter flow through the food web including total consumption, total export, total respiration, and total flows to detritus. PPR describes the total flow from trophic level I to sustain the consumption by trophic groups and fishery catches. FCI accounts for the percentages of matter flow that is generated by cycling. TE measures the percentage of matter flow transferred between two successive discrete trophic levels. These parameters quantify either the amount or the transfer efficiency of matter flow through food webs, and they are fundamentally important to ecosystem functioning (Woodward & Perkins, 2015).

Historical patterns of matter flow were reconstructed based on model M_4_ which reflected the ecosystem dynamics more closely to reality than the other models (Figure 2: Step 3). To detect deviations from the undisturbed condition, simulated biomasses and matter flow parameters were compared between M_4_ and M_0_ by the formula:

(3) $$Deviation=\left(M_{4}‐M_{0}\right)/M_{0}$$

Significance of deviations throughout the time period was detected by Cohen's d, where a value of >0.8 indicates a large effect, <0.2 a small effect, and 0.2–0.8 a middle effect (Cohen, 1988). A loess curve was fitted for each matter flow parameter as well as its deviation along the time period. SS values of the five Ecosim models were compared to estimate the overall contribution of each stressor. For each matter flow parameter, a generalized linear mixed model (GLMM) was constructed to disentangle the effects of multiple stressors. Simulated data of each parameter were extracted from the five Ecosim models (M_0_–M_4_) and combined to form a new data matrix containing matter flow parameter, year, and the three stressors. Since fish data were lacking for a yearly quantification of biological invasion, the three stressors were all treated as binary (presence/absence) variables. A global linear mixed effects model was constructed for each matter flow parameter, where the fixed part included the three stressors and interactions among them. A Gamma distribution with inverse link function was used for all models except for benthic algae biomass model where an inverse Gaussian distribution was used. A random intercept model was considered where year was treated as the random part considering duplications. To select the best fitted model, variables were removed one by one from the fixed part of the global model, while maintaining the random part (Zuur et al., 2013). Different models were compared by Akaike information criterion (AIC) (Akaike, 1974), and the final fitted model was determined as the model with the lowest AIC value, and all included variables were statistically significant.

Scenario forecasting was performed based on model M_4_, and four scenarios were considered. For comparison, scenario 1 was set to maintain a status of the latest (2015) condition, keeping all stressors unchanged. Scenario 2 was set to reduce TP to 0.05 mg/L, a status before 1970, considering eutrophication remediation. Scenario 3 was set to stop stocking (zero stocking) of planktivorous fish, while scenario 4 was double stocking. Regarding biological invasion, no scenario forecasting was considered since it is impractical to remove any introduced species out of the lake. Model M_4_ was performed for extending two decades for each scenario, and stressors were set to vary with fixed rates to reach the target values within the extended time frame (20 years). The forecasted values of scenarios 2–4 were compared with scenario 1 to detect the changes of matter flow parameters.

All statistical analyses were conducted with R version 3.6.1 (R Core Team, 2020).

## 3 RESULTS

**FIGURE 3:**
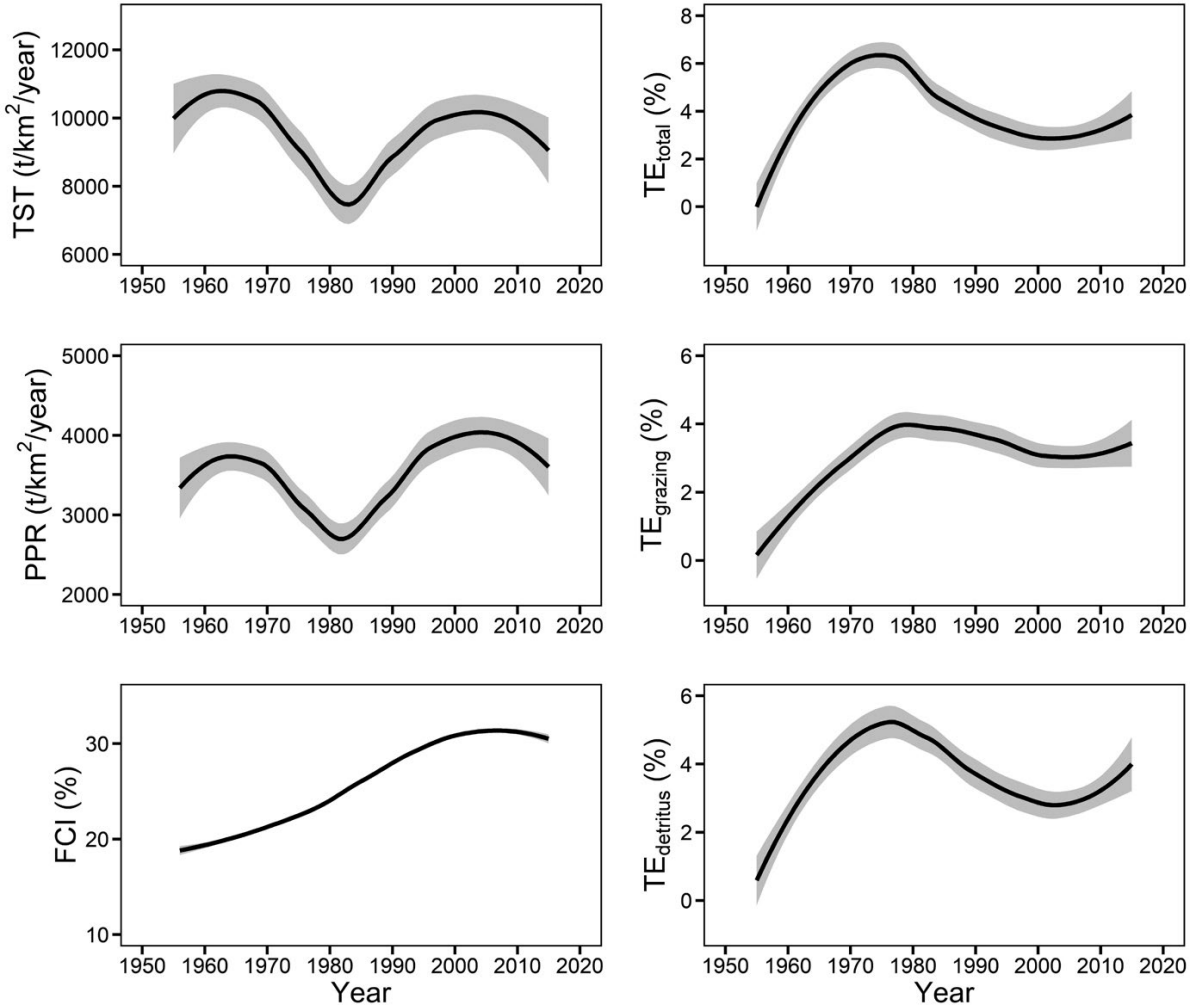
Loess curves (gray area, 95% CI) showing variations of matter flow parameters (based on model M_4_) during the 1950s‐2010s in Lake Dianchi food web

By comparing SS values between models M_1_‐M_4_ and M_0_, the total variance explained was M_1_ 15.8%, M_2_ 18.6%, M_3_ 64.1%, and M_4_ 68.1%, where M_4_ was the best fitted model. Matter flow parameters changed differently during the modeling period (Figure 3). Variations of TST and PPR showed quite similar hump‐shaped curves with two peaks occurring around the middle 1960s and middle 2000s, respectively. FCI increased monotonically, showing an s‐shaped curve. TE_total_, TE_grazing_, and TE_detritus_ changed similarly with a hump‐shaped curve and all peaked in the 1970s. Most parameters tended to change slowly or level off at the end of modeling period.

**FIGURE 4:**
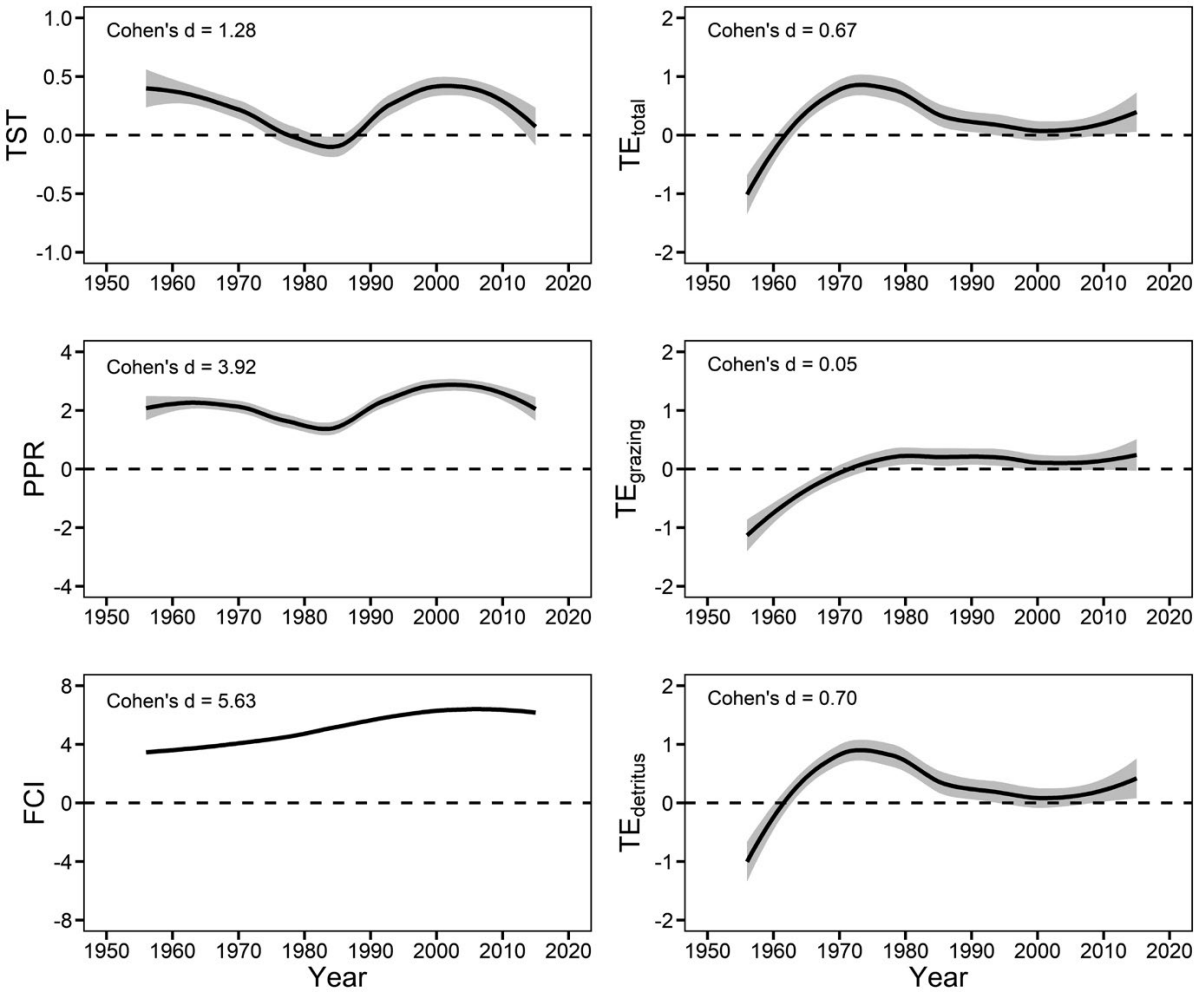
Loess curves (gray area, 95% CI) showing deviations of matter flow parameters from the natural condition (dash lines, models M_4_ versus. M_0_) during the 1950s‐2010s in Lake Dianchi

**FIGURE 5:**
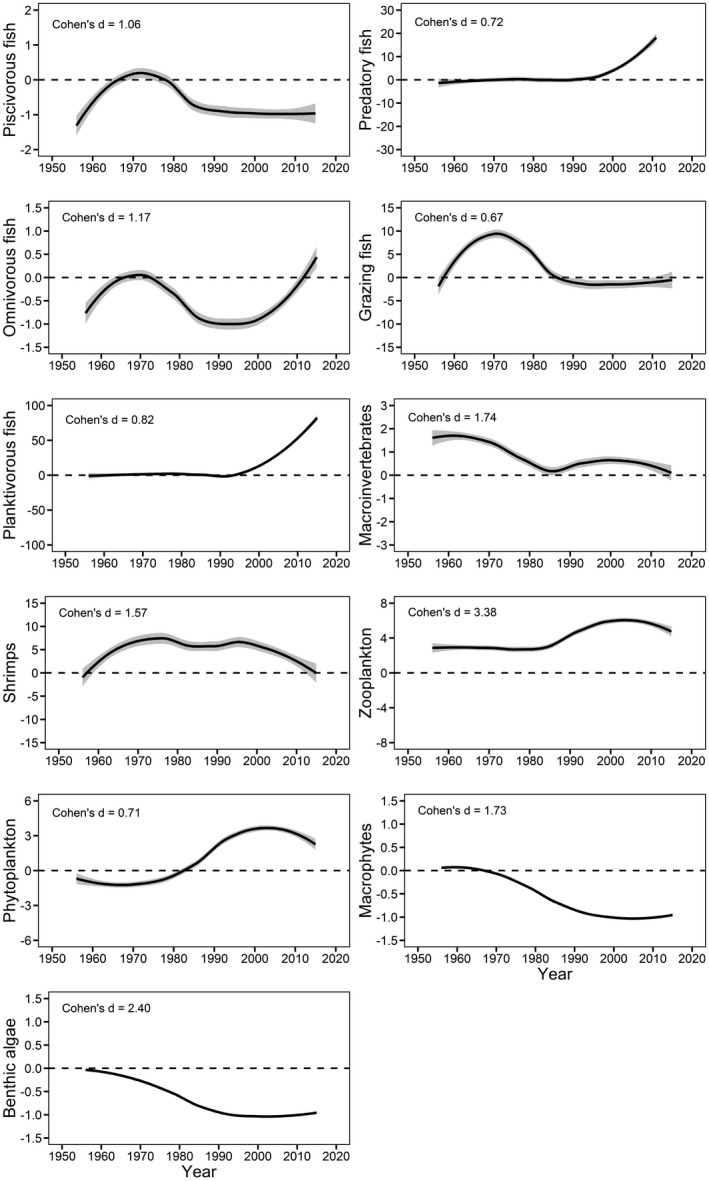
Loess curves (gray area, 95% CI) showing deviations of functional group biomasses from the natural condition (dash lines) during the 1950s‐2010s in Lake Dianchi

Most biomasses and matter flow parameters presented large deviations from the undisturbed condition (M_0_) as indicated by Cohen's d values (Figure 4, Figure 5). Biomasses of functional groups on the lower trophic levels had greater deviations than those on the higher trophic levels (Figure 5). Regarding matter flow parameters, TST, PPR, and FCI showed large deviations, while TE_total_ and TE_detritus_ middle and TE_grazing_ small deviations (Figure 4).

Biological invasion was the most important accounting for 49.4% of the total variance of M_4_, followed by eutrophication (15.9%) and biomanipulation (2.8%). The three stressors contributed to differing extents in explaining variances of biomasses and matter flow parameters (Table 3). Eutrophication and biological invasion were significantly, either severally or jointly, correlated with biomasses of all functional groups. Eutrophication played a stronger role on functional groups of top predator, pelagic food chain, and basal primary producers, while biological invasion had a larger effect on groups in the middle of food chains. Biomanipulation was of little importance and only included in planktivorous fish model. For matter flow parameters, both eutrophication and biological invasion were important and included in each model. Eutrophication was stronger in driving PPR and FCI, while biological invasion was more important on TST, TE_total_, and TE_detritus_, and both had equal roles on TE_grazing_. No interactions among the stressors were detected except for the planktivorous fish biomass model.

**TABLE 3:** Generalized linear mixed models^a^ for matter flow parameters of Lake Dianchi

Model	Estimate	*SE*	*p*
Piscivorous fish biomass			
Intercept	13.87	1.53	<.001
Eutroph	9.96	1.43	<.001
Predatory fish biomass			
Intercept	8.86	0.87	<.001
Eutroph	−5.03	0.90	<.001
BioInv	2.00	0.46	<.001
Omnivorous fish biomass			
Intercept	1.00	0.09	<.001
Eutroph	−0.47	0.10	<.001
BioInv	1.30	0.13	<.001
Grazing fish biomass			
Intercept	81.58	24.18	<.001
Eutroph	286.50	58.18	<.001
BioInv	−360.94	52.99	<.001
Planktivorous fish biomass			
Intercept	11.12	0.96	<.001
Eutroph	−8.99	0.96	<.001
BioInv	0.65	0.20	.001
Bioman	−0.32	0.10	.002
BioInv:Bioman	−0.49	0.22	.024
TST			
Intercept	8,044.22	232.58	<.001
Eutroph	833.00	147.35	<.001
BioInv	1,081.23	120.31	<.001
PPR			
Intercept	0.0009	0.00002	<.001
Eutroph	−0.0006	0.00001	<.001
BioInv	−0.0001	0.00001	<.001
FCI			
Intercept	0.24	0.002	<.001
Eutroph	−0.19	0.002	<.001
BioInv	−0.01	0.0003	<.001
Shrimps biomass			
Intercept	4.84	0.19	<.001
Eutroph	−1.15	0.18	<.001
BioInv	−2.52	0.09	<.001
Macroinvertebrates biomass			
Intercept	0.07	0.002	<.001
BioInv	−0.03	0.001	<.001
Zooplankton biomass			
Intercept	0.11	0.001	<.001
Eutroph	−0.10	0.001	<.001
BioInv	−0.001	0.000	<.001
Macrophytes biomass			
Intercept	0.0004	0.0001	<.001
Eutroph	0.0005	0.0001	<.001
Phytoplankton biomass			
Intercept	0.16	0.02	<.001
Eutroph	−0.13	0.02	<.001
Benthic algae biomass			
Intercept	0.91	0.37	0.013
Eutroph	5.80	0.90	<.001
TE_total_			
Intercept	0.36	0.02	<.001
Eutroph	0.09	0.01	<.001
BioInv	−0.17	0.01	<.001
TE_grazing_			
Intercept	0.37	0.03	<.001
Eutroph	0.16	0.02	<.001
BioInv	−0.17	0.02	<.001
TE_detritus_			
Intercept	0.37	0.02	<.001
Eutroph	0.08	0.01	<.001
BioInv	−0.17	0.01	<.001

Abbreviations: Eutroph, Eutrophication; BioInv, biological invasion; Bioman, biomanipulation.

^a^ Only the fixed part of each model is shown.

**FIGURE 6:**
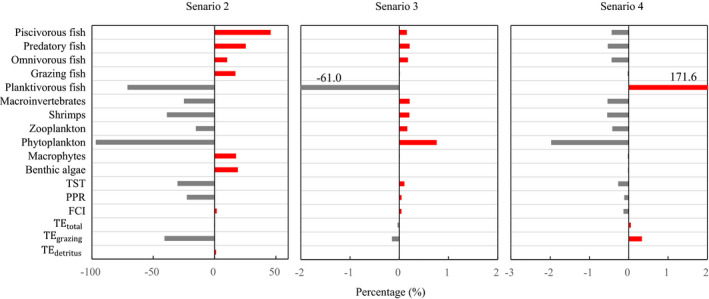
Changes in functional group biomasses and matter flow parameters under three scenarios. Scenario 2, reducing TP to 0.05 mg/L; Scenario 3, zero stocking of planktivorous fish; Scenario 4, double stocking of planktivorous fish

Scenario forecasting showed that reducing TP to 0.05 mg/L could lead to great changes of the lake food web (Figure 6, scenario 2). Biomass of phytoplankton would be reduced by 97%, followed by planktivorous fish, shrimps, macroinvertebrates, and zooplankton. In contrast, biomass of piscivorous fish would be increased by 46%, followed by predatory fish and the rest functional groups. Among matter flow parameters, TE_grazing_ would be reduced by 41%, followed by TST and PPR, while the others would be little changed (<2%). Both zero and double stocking of planktivorous fish could lead to little change (<2%) of the food web except for biomass of themselves group (Figure 6, scenario 3 and 4).

## 4 DISCUSSION

### 4.1 Effects of multiple stressors

Our results showed that the matter flow pattern of Lake Dianchi changed greatly during the 1950s–2010s, indicating that the ecosystem was highly dynamic in this period. Variations of most ecosystem parameters coincided with the presence of major anthropogenic stressors (Li et al., 2014), indicating that the dynamicity was largely attributed to these multiple stressors. We found that TST, PPR, and FCI had larger deviations than the three TE parameters, indicating that multiple stressors exerted greater effects on the quantity of matter flow than trophic transfer efficiency in the lake food web. Previous studies showed that transfer efficiencies varied greatly (between 2% to 50%) across space and time, and might be sensitive to environmental changes (Post, 2002). In this study, TE_detritus_ had a larger deviation than TE_grazing_, suggesting that trophic transfer efficiency along the detritus food chain might be more sensitive to multiple stressors than that along the grazing food chain.

We found that biological invasion was the most important in driving the matter flow in Lake Dianchi food web. It has been generally recognized that biological invasion can exert detrimental effects on ecosystem functioning to various extents (Little & Altermatt, 2018; Strayer, 2012). In the present study, biological invasion was included to explain variances of biomasses of most functional groups and all matter flow parameters. Introduction of non‐native fish species has greatly changed the fish assemblage structure of Lake Dianchi, leading a shift from native to non‐native dominated assemblages in the past decades (Chen et al., 2001; Yuan et al., 2010). Both the quantity and transfer efficiencies of matter flow were greatly shaped by biological invasion in the lake food web. It seemed that alterations of community structure could further lead to changes in ecosystem functioning to a large degree.

The possible explanations for non‐native fish species driving the matter flow of the lake food web were as follows. First, most non‐native fishes were translocated from the lowland region of the Yangtze River, characterized by rapid growth and early maturation. By contrast, native species in the highland region are regarded as slow growth and late maturation attributing to the high‐elevation environments (Chen et al., 2009; Liu et al., 2017). The fast growth rates together with probably high metabolic rates of non‐native species might enable them to accumulate biomass and transfer matter and energy more quickly than native species. Second, the role of non‐native species in shaping matter flow pattern might be strengthened by continuous and repeatedly introduction as well as lack of effective recruitment of native species. The initially introduced species, the four Chinese carps (i.e., *Mylopharyngodon piceus* (Richardson), *Ctenopharyngodon idella* (Valenciennes), *Hypophthalmichthys molitrix,* and *H*. *nobilis*), are river‐lake migratory fishes, and they cannot reproduce in isolated lakes such as Lake Dianchi. To improve fishery production, these species were continuously introduced into the lake. Simultaneously, some small‐sized species such as *Pseudorasbora parva* (Temminck & Schlegel), *Rhodeus ocellatus* (Kner), and *Micropercops swinhonis* (Günther) were brought into the lake accidentally. These small‐sized fishes can grow fast and some of them swallow eggs of native species, exerting great pressures on the local fish assemblage (Chen et al., 1998, 2001). In addition, recruitment of native species might be rare since most of them are now confined to the tributaries where their populations are quite small (Chen et al., 2001).

Our results showed that eutrophication played an important role in shaping matter flow pattern of the lake food web. It has been generally acknowledged that lake eutrophication can promote the pelagic primary production to a great extent but exert strong detrimental effects to benthic primary production (Schindler, 2012; Smith, 2003). In the present study, both the pelagic (phytoplankton) and benthic (macrophytes and benthic algae) primary producers were found to be mainly driven by eutrophication. Effects of eutrophication could be transferred to the higher trophic levels through the bottom‐up effects as well as by altering the physical–chemical environments (Carpenter et al., 2001; Vadeboncoeur et al., 2003; Vasconcelos et al., 2019). We found that eutrophication played a stronger role in shaping biomasses of functional groups on the pelagic food chain than those on the benthic food chain, indicating that eutrophication‐induced bottom‐up effects might be more effectively transferred in the former food chain. In addition, most biological communities might be suppressed when a lake became hypereutrophicated, leading to decreases in total matter flow of the whole ecosystem (Wang et al., 2020). Similarly, we found that TST in the lake food web was decreasing after the early 2000s when the lake stepped into a hypereutrophic state. Previous studies showed that eutrophication could also shape the benthic food web by strengthening the benthic‐pelagic coupling (Genkai‐Kato et al., 2012; Vadeboncoeur et al., 2001; Wang et al., 2020) and consequently improve the cycling of matter in the whole lake food web. It was also the case in Lake Dianchi as we found an increasing tendency of FCI which was mainly driven by eutrophication in the past decades. In addition, eutrophication could shape the matter flow pattern by changing trophic transfer efficiencies as our results showed that it was included in all the three TE models. Eutrophication can cause harmful algal blooms (usually cyanobacteria blooms) to various extents, which cannot be consumed by zooplankton and even generate toxins harmful to aquatic biota, leading to decreases in transfer efficiencies of matter and energy (Alexander et al., 2017). In Lake Dianchi, heavy cyanobacteria blooms occur during April‐October, a time period much longer than that in the Yangtze lowland lakes (Li et al., 2014; Wang et al., 2020). Consequently, eutrophication probably contributed to a large extent to the decreases of trophic transfer efficiencies in Lake Dianchi after it was eutrophicated.

We found that biomanipulation, that is, phytoplankton control by planktivorous fish stocking, was of little importance in shaping the matter flow pattern of Lake Dianchi. Not as we expected, stocking of planktivorous fishes had little effect on phytoplankton biomass. Similar results were also found in the Yangtze lowland lakes (Wang et al., 2008). Although planktivorous fish can feed on cyanobacteria, its assimilation rate is quite low (e.g., less than 4% for silver carp) (Wang et al., 2015), and only a small proportion of nutrients could be removed from the waterbody along with fish catch. It seemed that biomanipulation did not work well in the large hypereutrophic lake, where nutrients induced bottom‐up effects held an overwhelming role in driving primary production.

Previous studies showed that additive stressor effects were common in freshwater ecosystems(Jackson et al., 2016). In this study, little interactions among biological invasion, eutrophication, and biomanipulation were detected in generalized linear mixed models, indicating that effects of these stressors on matter flow of the lake were mainly additive. We assumed that this finding was related to the integrative nature of our responsible variables, that is, matter flow parameters. The addressed stressors in this study could affect matter flow not only by strong direct effects (e.g., predation) but also by complex indirect effects (e.g., trophic cascades), as those in many other ecosystems (Wootton, 1994, Guimarães et al., 2017). Such mechanistic complexity of stressor–response relationships might obscure the interactions among multiple stressors (Gieswein et al., 2017). In addition, whether the stressors’ effects are additive or not is dependent on the stressor combinations as well as the responsible variables addressed (Jackson et al., 2016). Although biological invasion and eutrophication occur simultaneously in many freshwater bodies, how do they interact to affect other ecosystem functioning remains largely unclear.

### 4.2 Implications for ecosystem management

Understanding the effects of multiple stressors on ecosystem functioning is helpful for effective management of freshwater ecosystems (Reid et al., 2019; Woodward & Perkins, 2015). Here, we disentangled the effects of biological invasion, eutrophication, and biomanipulation on matter flow of a lake ecosystem. Since matter flow reflects aggregate processes at population and community scales (e.g., production, trophic transfer) (Barnes et al., 2018), our results could provide important implications regarding ecosystem multifunctionality and holistic management in lakes. Tackling interactions among multiple stressors is challenging for ecosystem management (Brown et al., 2013). In the present study, we found that the effects of addressed stressors were mainly additive, indicating that they acted independently of each other. Therefore, ecosystem management of the lake might not need to pay much attention on the interactions of multiple stressors. Mitigating effects of individual stressor, for example, biological invasion or eutrophication, might lead to good results in ecological restoration.

Although biological invasion might play a stronger role than eutrophication in the lake ecosystem, remediation of eutrophication is of first consideration given that good water quality provides fundamentally physical–chemical environments for biological restoration (McCrackin et al., 2017; Schindler, 2012). Scenario forecasting showed that improving water quality could largely reduce biomasses of phytoplankton and planktivorous fish, and increase biomasses of piscivorous and predatory fish. It should be acknowledged that restoring an ecosystem back to a relatively good condition is much harder than we destroy it (Jeppesen et al., 2012; Raimundo et al., 2018). The studied lake ecosystem might not be actually recovered as forecasted when we reduced TP of lake water to 0.05 mg/L, since other nutrients such as nitrogen should also be controlled, and more importantly, the total water quality should be improved. It is not necessary to discuss here how to restore eutrophicated lakes, which has been thoroughly addressed in a number of publications (e.g., Jeppesen et al., 2012; McCrackin et al., 2017; Schindler, 2012).

Mitigating the effects of biological invasion is of great importance considering that non‐native species played the strongest role in driving the changes in matter flow of the lake. However, it is impractical, and to some extent unnecessary, to remove all introduced species out of the lake, considering that non‐native species dominate the present‐day fish assemblage both in species richness and in fish catch (Chen et al., 2001; Ye et al., 2015; Yuan et al., 2010). We have to accept this “new” and “hybrid” ecosystem. Although we did not carry out any scenario forecasting related to control of biological invasion, applying appropriate measures to mitigate its effects is hopefully to improve the ecosystem to certain degrees. In this regard, several possible measures were put forward as follows. The first is to carry out systematic assessments of the effects of non‐native species in the lake and even better in the highland region and to stop any introduction of non‐native species regardless of any purposes. The second is to make more efforts to conserve the native species especially their habitats, considering that many of them are now confined in the tributaries and in face of habitat loss (Chen et al., 2001). The third is to strengthen recruitment of native species either from natural populations or by artificial breeding.

Regarding biomanipulation, scenario forecasting showed that the lake ecosystem would be little changed under both zero stocking and double stocking of planktivorous fish. This finding was consistent with the results of generalized linear mixed models, indicating that stocking of planktivorous fish was probably not an efficient measure to control algal biomass nor effective to improve the ecosystem health in the hypereutrophic lake. Combined with other studies in the Yangtze region (Wang et al., 2008), we suggested that such biomanipulation measure might not be applicable in large, heavily eutrophicated waterbodies. However, the measure might work in small, slightly eutrophicated waterbodies where nutrients could be well controlled to ensure an overcome of top‐down effects on bottom‐up effects (Jeppesen et al., 2012). The effectiveness and efficiency of stocking planktivorous species as a biomanipulation measure should be systematically assessed, considering that there is an increasing tendency to apply it in ecological restoration planning and engineering in eutrophicated waterbodies.

Although we included the major stressors in our Ecosim models, other stressors such as habitat loss, fishery production, and climate change were also important in shaping matter flow pattern of the lake to certain degrees (Li et al., 2014). For effective restoration of the lake ecosystem, it was important to apply corresponding measures to cope with these stressors. Regarding ecological restoration of the lake, we suggested to adopt a holistic strategy with focus on biodiversity and ecosystem functioning, and to systematically regulate the ecosystem to a more healthy status (Palmer et al., 2014).

### 4.3 Limitations

We acknowledged three major limitations in the present study. The first limitation derived from limited data. In this study, biological data were compiled for every decade due to lack of yearly data, which led to uncertainties in model calibration and prediction to certain degrees. Also, this limitation was shown in the GLMMs where the stressors were treated as binary (presence/absence) instead of continuous variables. The second limitation was that we did not consider the input and output from tributaries of the lake in food web modeling. This might decrease the model efficiency and predictive ability. The third limitation was that stressors were imbalancedly represented in the Ecosim models, where biological invasion was represented by five nodes (i.e., five invasion groups) and other stressors by one. Consequently, the role of biological invasion might be potentially overestimated. Hopefully, these limitations can be mitigated in further studies as more detailed data are collated. In addition, the methodology we put forward might be applicable in other freshwater ecosystems with long‐term monitoring data.

## 5 CONCLUSION REMARKS

In the present study, we disentangled the effects of multiple stressors on matter flow in a lake food by integrating Ecosim models with GLMMs. Environmental stressors, such as biological invasion, operating on trophic transfer efficiency played a strong role in shaping matter flow in lake food webs. Our findings provided new insights into understanding the mechanistic links between anthropogenic stressors and ecosystem functioning, and had important implications for conservation and restoration of lake ecosystems. Moreover, we provided a methodology combining ecosystem modeling with linear regression into exploration of stressor‐ecosystem functioning relationships, although our Ecosim models were constructed and calibrated with limited data, leading to some uncertainties in model predictions.

## CONFLICT OF INTEREST

The authors declare no conflicts of interest.

## AUTHOR CONTRIBUTION

**Shuran Wang:** Data curation (equal); Formal analysis (equal); Investigation (lead); Methodology (equal). **Xueqin Liu:** Conceptualization (lead); Data curation (equal); Formal analysis (lead); Funding acquisition (lead); Methodology (equal); Writing‐original draft (lead). **Yong Liu:** Data curation (supporting); Funding acquisition (supporting); Project administration (supporting). **Hongzhu Wang:** Conceptualization (supporting); Formal analysis (supporting); Methodology (supporting).

## Supporting information

Supplementary Material

## Data Availability

The data that support the findings of this study are available at Dryad digital repository (https://doi.org/10.5061/dryad.7m0cfxptr).
