# Supplementary material for: Disentangling effects of multiple stressors on matter flow in a lake food web
Source: Ecol Evol. 2021 Jun 21;11(14):9652–64. doi: 10.1002/ece3.7789 (PMC8293722; doi:10.1002/ece3.7789)
Supplement: Supplementary file 1 — Supplementary Material [file ECE3-11-9652-s001.docx]

Table S1 List of references citied for biological data collations used for food web analyses of Lake Dianchi

| Biological groups | 1950s | 1960s | 1970s | 1980s | 1990s | 2000s | 2010s |
| --- | --- | --- | --- | --- | --- | --- | --- |
|  | 1950-1957 | 1958-1969 | 1970-1979 | 1980-1989 | 1990-1999 | 2000-2009 | 2010-2016 |
| Phytoplankton | Li et al. (2014), Dong (2014) | - | - | Li et al. (2014), Qian et al. (1985) | Li et al. (2014), Dong (2014) | Dong (2014), Zhang et al. (2006), Dai et al. (2012), Guo and Han (2012), Huang (2012) | Li et al. (2014), Liu et al. (2016), Wang et al. (2016), Wang et al. (2020) |
| Benthic algae | Li et al. (1963) | - | - | - | - | Pei et al. (2008) | Yang et al. (2013) |
| Macrophytes | Yu et al. (2000), Zhou et al., (2013) | Yang et al. (2004) | Li (1980) | Dai (1985), Yang et al. (2004) | Yu et al. (2000) | Yang et al. (2004), Chen et al. (2003), Shen et al. (2010) | Xiang (2014), Wang et al. (2020) |
| Zooplankton | Li et al. (1963) | - | - | Wang Z. (1985), Huang and Zhao (1992), Dong (2015) | Peng (1995), Dong (2015) | Sun (2010), Dong (2015) | Liu et al. (2016), Wang et al. (2020) |
| Macroinvertebrates | Zhang and Qi (1949), Zhang (1948) | - | Zhang and Wu (1983)* | Wang L. (1985)* | Li et al. (2014), Luo et al. (2006) | Wang et al. (2002), Wang (2012) | Wang et al. (2011), Wang et al. (2018) |
| Fish | Chen (1991), Chen et al. (2001), He and Liu (1985), Gao et al. (1981) | Wang (1988), Chen (1991), He and Liu (1985), Gao et al. (1981) | Chen (1991), He and Liu (1985), Chen et al. (2001), Chen (1981) | He and Liu (1985), Wang (1988), Chen et al. (2001) | Chen et al. (1998), Yang et al. (1994), Statistics Bureau of Kunming (1991-2015) | Chen et al. (1998), Yuan et al. (2010), Ye et al. (2015), Statistics Bureau of Kunming (1991-2015) | Wang et al. (2020), Statistics Bureau of Kunming (1991-2015) |

-, data was unavailable. *, only species list was available.

Chen K., Li W., Wu Q. and Qiang S. 2003. Impacts of cyanobacteria on the growth of submerged macrophytes, Dianchi Lake. Journal of Lake Sciences, 15(4), 364-368.

Chen P. 1981. Waterbody status and fishery in Yunnan Dianchi Lake. Freshwater Fisheries, 6, 6-8.

Chen Y., Yang J. and Li Z. 1998. The diversity and present status of fishes in Yunnan Province. Chinese Biodiversity, 6(4), 272-277.

Chen Y. 1991. Fish resources and its utilization and conservation in Yunnan Province. Nature Resources, 1, 25-33.

Chen Z., Yang J., Su R. and Chen X. 2001. Present status of the indigenous fishes in Dianchi Lake, Yunnan. Biodiversity Science, 9, 407-413.

Dai G., Li J. Li L. and Song L. 2012. The spatio-temporal pattern of phytoplankton in the north basin of Lake Dianchi and related environmental factors. Acta Hydrobiologica Sinica, 36(5), 946-956.

Dai Q. 1985. The ecological characteristics of the aquatic vegetations in the lake of Fuxianhu, Erhai and Dianchi in Yunnan Plateau. Acta Ecologica Sinica, 5(4), 324-335.

Dong Y. 2014. Research and development of algae in the nine plateau lakes in Yunnan. Environmental Science Survey, 33(2), 1-8.

Dong Y. 2015. Review of research on zooplankton of the nine plateau lakes in Yunnan Province. Environmental Science Survey, 34(5), 5-10.

Gao L., Zhuang D., Zhang K. and Guo Q. 1981. A preliminary survey on quantity variance of commercial fishes in lakes of Yunnan Plateau. Chinese Journal of Zoology, 1, 31-34.

Guo Y. and Han Y. 2012. A study on the influence of large-scale water hyacinth planting on the phytoplankton in Dianchi Lake. Environmental Science Survey, 31(6), 47-51.

He J. and Liu Z. 1985. An analysis of the causes of fish quantity variance from changes of the fish fauna in Yunnan Dianchi Lake. Journal of Yunnan University, 7 (Supplement), 29-36.

Huang H. and Zhao J. 1992. Evaluation of the pollution in Lake Dianchi with zooplankton as indicator. Chinese Journal of Environmental Science, 13(3), 33-36.

Huang J. 2012. An analysis of phytoplankton change trend in Dianchi Lake. Environmental Science Survey, 31(5), 35-37.

Li G., Li L., Pan M., Xie Z., Li Z., Xiao B., Liu G., Chen J. and Song L. 2014. The degradation cause and pattern characteristics of Lake Dianchi ecosystem and new restoration strategy of ecoregion and step-by-step implementation. Journal of Lake Sciences, 26, 485-496.

Li H. 1980. A study on the lake vegetation in the Yunnan Plateau. Acta Botanica Yunnanica, 2(2), 113-141.

Liu C., Yu Y., Wang R., Song D., Yang S. and Wang Z. 2016. The plankton diversity of Dianchi Lake. Journal of West China Forestry Science, 45(1), 74-80.

Ley S., Yu M., Li G., Tseng J., Chen J., Kao B. and Huang H. 1965. Limnological survey of the lakes of Yunnan plateau. Oceanologia et Limnologia Sinica, 5(2), 87-114.

Luo M., Duan C., Shen X. and Yang L. 2006. Environmental degradation and loss of species diversity in Dianchi Lake. Marine Fisheries, 28(1), 71-78.

Peng Q. 1995. A survey on zooplankton in Dianchi Lake. Reservoir Fisheries, 6, 22-26.

Pei G., Liu G. and Hu Z. 2008. Benthic algal communities distribution in the littoral zone of Yunnan Plateau lakes. Journal of Wuhan Botanical Research, 26(4), 373-378.

Qian C., Deng, X., Wang, R. and Xu J. 1985. A study on the Dianchi Lake algal flora. Journal of Yunnan University, 7 (Supplement), 9-28.

Shen Y.-Q., Wang H.-J. and Liu X.-Q. 2010. Aquatic flora and assemblage characteristics of submerged macrophytes in five lakes of the central Yunnan Province. Resource and Environment in the Yangtze Basin, 19, 111-119.

Statistics Bureau of Kunming. 1991-2015. Kunming Statistical Yearbook. China Statistics Press. Beijing.

Sun C. 2010. The study on the community structure and the variation of population quantity in Dianchi Lake. Master thesis, Yunnan University.

Wang H., Yang S., Fang S., Yu F., Feng W. and Liu L. 2016. Canonical correspondence analysis of relationship between characteristics of phytoplankton community and environmental factors in Dianchi Lake. China Environmental Science, 36(2), 544-552.

Wang S.C., Liu X., Liu Y. and Wang H. 2018. Contrasting patterns of macroinvertebrates inshore vs. offshore in a plateau eutrophic lake: Implications for lake management. Limnologica, 70, 10-19.

Wang S.C., Liu X., Liu Y. and Wang H. 2020. Benthic-pelagic coupling in lake energetic food webs. Ecological Modelling, 417, 108928.

Wang X. 1988. Ecological economic benefit of fisheries in Dianchi Lake. Ecological Economy, 1, 45-46.

Wang Z. 1985. The basic survey of the zoo-plankton in Yunnan Dian-Chi Lake. Journal of Yunnan University, 7 (Supplement), 53-72.

Wang L. 1985. A research of biger invertebrates in Yunnan Dianchi Lake. Journal of Yunnan University, 7 (Supplement), 73-84.

Wang L., Xu X., Zhou W. and Xiao H. 2002. A study on the zoobenthos in Macunwan and Haidongwan region of Dianchi Lake Yunnan. Journal of Yunnan University, 24(2), 134-139.

Wang H. 2012. Surveys on aquatic biota in lakes on the Yunnan-Guizhou Plateau. Project report.

Xiang X. 2014. A study on higher plants and their communities in wetlands around Dianchi Lake. Master thesis, Yunnan University.

Yang K., Dong J., Guo L. and Li G. 2013. The population structure and the distributing characteristics of *Cladophora* in the littoral zone of Dianchi Lake. Journal of Hydroecology, 34(3), 8-16.

Yang Z., Zhang X. and Liu A. 2004. Study on Aquatic Vegetation Change in Dianchi Lake, Journal of Southwest Forestry College, 24(1), 27-30.

Yang J., Chen Y. and He Y. 1994. Studies on fish diversity in plateau lakes of the central Yunnan. Chinese Biodiversity, 2(4), 204-209.

Ye S., Lin M., Li L., Liu J., Song L. and Li Z. 2015. Abundance and spatial variability of invasive fishes related to environmental factors in a eutrophic Yunnan Plateau lake, Lake Dianchi, southwestern China. Environmental Biology of Fishes, 98, 209-224.

Yuan G., Ru H.-J. and Liu X.-Q. 2010. Fish diversity and fishery resources in lakes of Yunnan Plateau during 2007-2008. Journal of Lake Sciences, 22, 837-841.

Yu G., Liu Y., Qiu C. and Xu X. 2000. Macrophyte succession in Dianchi Lake and relations with the environment. Journal of Lake Sciences, 12(1), 73-80.

Zhang Q. and Wu T. 1983. Dianchi Lake Pollution and Hydrobios. Yunnan People Publishing House, Kunming City.

Zhang X. and Qi Z. 1949. Yunnan freshwater mollusks and its new species. Contributions from the Institute of Zoology National Academy of Peiping, 5(5), 219-220.

Zhang X. 1948. The research on Kunming Lake and its animals. Contributions from the Institute of Zoology National Academy of Peiping, 4, 11-24.

Zhang M., Li Y. and Wang R. 2006. Dynamic variation for the species of phytoplankton in Dianchi Lake, China, Journal of Yunnan University, 28(1), 73-77.

Zhou H., Kong D., Fan Y., Yang F. and Chen J. 2013. Research progress of aquatic macrophytes in Dianchi Lake, Environmental Science & Technology, 36(12M), 187-194.


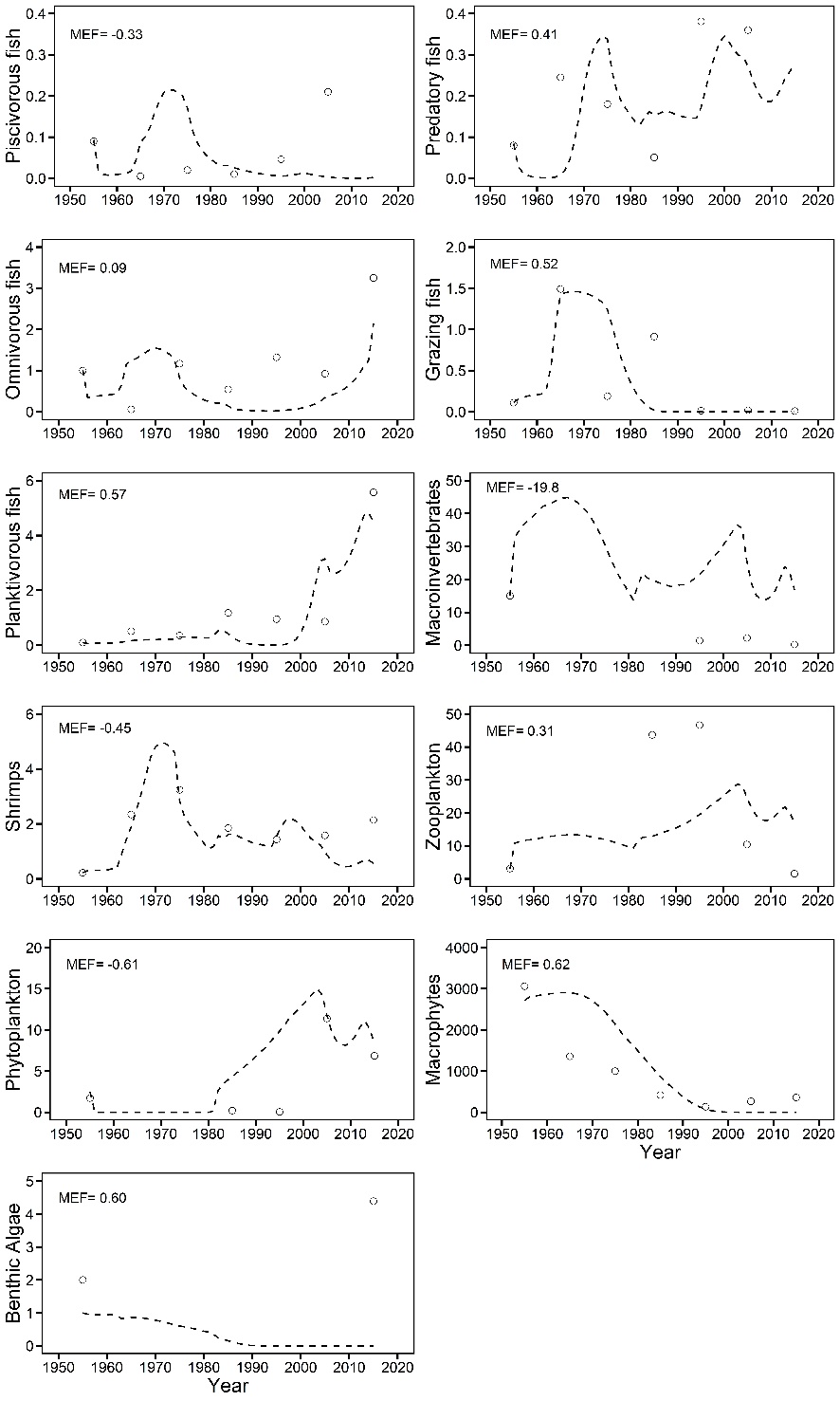


Figure S1 Simulated (dashed line) vs. observed (hollow circle) biomass of each functional group in the best fitted model (M4). MEF is the modelling efficiency index. A value of MEF close to 1 indicates a perfect fit, 0 indicates no better than using the average, and a negative value indicates a poor fit.
